# A Novel and Practical Protocol for Three-Dimensional Assessment of Alveolar Cleft Grafting Procedures

**DOI:** 10.1177/10556656221074210

**Published:** 2022-03-02

**Authors:** Celine C. Stoop, Nard G. Janssen, Timen C. ten Harkel, Antoine J. W. P. Rosenberg

**Affiliations:** 1 8124 Utrecht University Medical Center, Utrecht, the Netherlands

**Keywords:** cleft lip and palate, alveolar cleft, cone beam computed tomography, segmentation

## Abstract

**Objective:**

To evaluate the reproducibility and accuracy of a new, easy-to-use volumetric assessment of the alveolar cleft.

**Design:**

Twelve cone-beam computed tomography (CBCT) datasets of patients with a unilateral cleft lip, alveolus, and palate were evaluated by two investigators. Residual alveolar cleft calcified volume one year after surgery was analyzed by using standardized landmarks to determine the borders of the cleft defect and semi-automatically segment the alveolar cleft defect.

**Results:**

The Dice-coefficient between observers for the segmented preoperative alveolar cleft defect was 0.81. Average percentage of residual alveolar cleft calcified material was 66.7% one year postoperatively.

**Conclusions:**

This study demonstrates a reliable and practical semi-automatic three-dimensional volumetric assessment method for unilateral clefts using CBCT.

## Introduction

Cleft lip and palate incidence is rather common and is found in every 1.5:1000 births.^1^ The alveolar cleft defect is the most common congenital bone deformity of the craniofacial skeleton. The surgical treatment protocol for patients with cleft lip, alveolus, and palate in most cleft teams worldwide includes secondary alveolar bone grafting (SABG).^2^ This treatment is usually performed at the end of the mixed dentition stage, between the ages of nine and twelve years.

The primary outcomes of SABG are fusion of the segments of the maxillary arch for continuity, closure of the oronasal fistula, providing a bony construct to facilitate the eruption of the canine, and lateral incisor, with an uninterrupted arch and improving nasolabial support and symmetry of facial esthetics.

Several two- and three-dimensional measurement methods have been proposed to assess alveolar cleft volume. Mainly, two-dimensional radiological assessment was carried out using standardized scales.^3–5^ However, three-dimensional measurement methods seem to be more precise than two-dimensional methods.^6^

Different problems in various cleft assessment methods are encountered. The first problem is establishing cleft defect landmarks because of the differences in morphology and complexity of the boundaries of the defect. This is especially relevant for the palatal boundary of the alveolar cleft defect. In most patients with a complete alveolar cleft, the palatal part is difficult to establish.

Secondly, the growing skeleton in combination with the eruption of the permanent dentition often poses a problem.^7^ By comparing preoperative scans with postoperative scans, it is difficult to accurately match the changing cleft defect and measure the new required bone volume.

Cone-beam computed tomography (CBCT) can be a precise and effective modality for three-dimensional assessment of alveolar cleft volume.^8^ A large and increasing number of studies report on different methods using CBCT scans to assess this bone defect. However, in many cases the bony defect is hard to define in both preoperative and postoperative scans. These studies do not make use of superimposition of preoperative and postoperative scans and thereby making it virtually impossible to distinguish between preexistent and reconstructed maxillary bone.

The amount of digital image analysis software of semi-automatic segmentation methods for volumetric assessment of alveolar clefts rises. A disadvantage of these methods is that it has been proven difficult to create both a reliable and practical method to measure and compare alveolar cleft defects before and after reconstruction. Semi-automated segmentation protocols are often limited due to the different definition of the borders of the cleft defect. Especially the palatal bony boundary is not specified in cases with complete alveolar clefts.^7,9^

Combining semi-automatic segmentations, with well-defined borders and precise standardized anatomic landmarks might improve the reproducibility of the assessment method.

The aim of this study was to evaluate the reproducibility and accuracy of a new, easy-to-use volumetric assessment of the alveolar cleft.

## Methods

### Subjects

Twelve randomly selected patients with a unilateral cleft lip, alveolus, and palate were selected that underwent SABG. All patients included were operated on between June 2018 and July 2020 and were operated on at the Utrecht University Medical Center by a single surgeon.

Preoperative CBCT scans were taken 1 to 4 weeks prior to surgery and 12 months postoperatively (range 10-15 months) to evaluate the volume of the cleft area. Scans of all patients were taken according to a standard CBCT scanning procedure (NewTom VGi evo). The image acquisition parameters included settings of 89.9 or 110 kV with a range of 7.2 to 28.35 mAs (pulsed mode). The field of view was 78, 82.8 or 149.8 cm and the voxel size was 0.3 to 0.35 mm.

The CBCT scans were processed with BrainLab Elements software (BrainLab AG).

### Anatomical Landmarks

**Figure 1.**
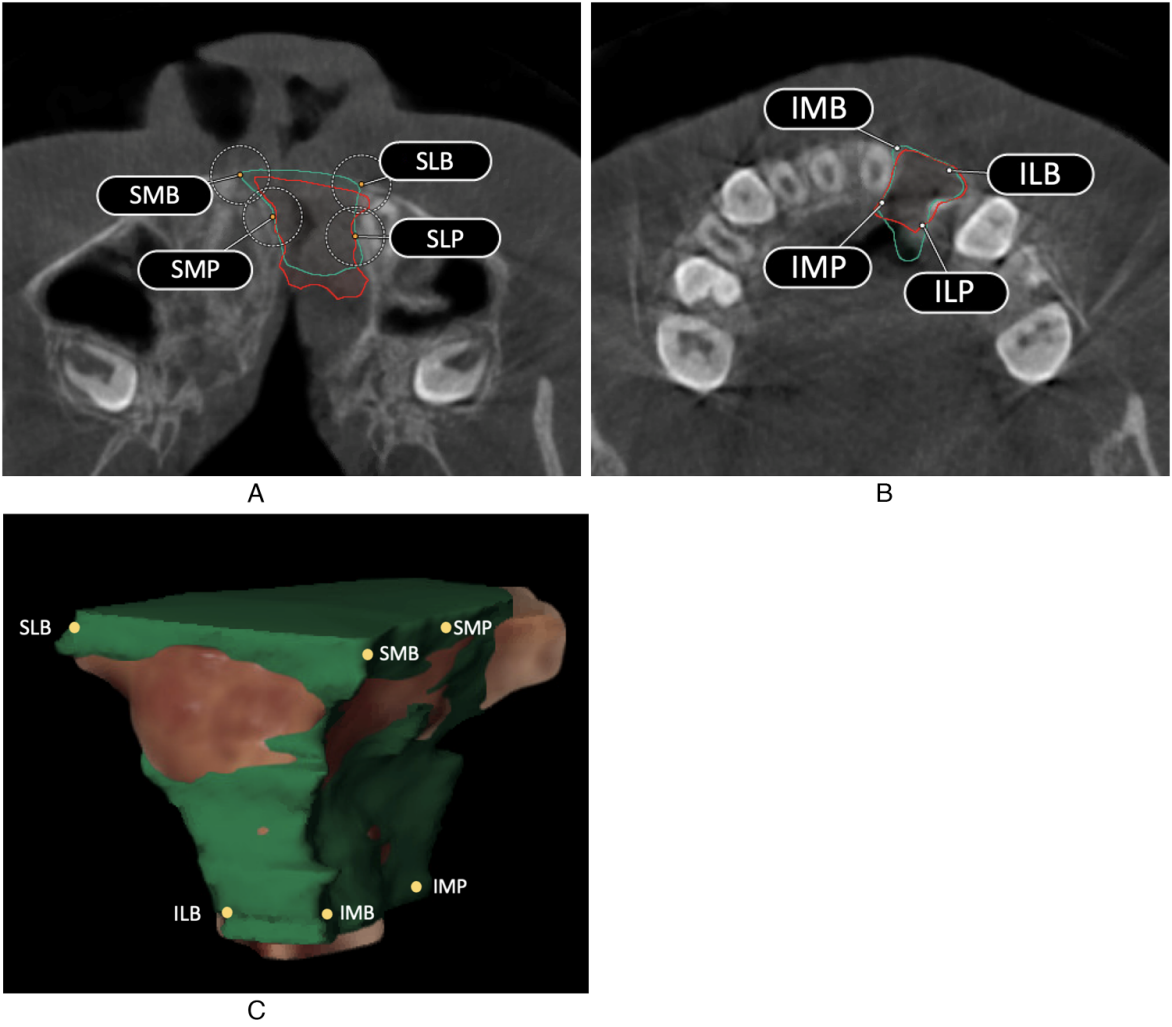
Display of the landmarks in the alveolar cleft. (A) Axial CBCT slice of most cranial (superior) part of the alveolar cleft defect. SMB is the most medial-buccal border of the alveolar cleft. SLB is the most lateral-buccal border of the alveolar cleft. SMP was placed 8 mm in palatal direction from landmark SMB. SLP was placed 8 mm in palatal direction from landmark SLB. (B) Axial CBCT slice of the most caudal (inferior) part of the alveolar cleft defect. IMB is the most medial-buccal border of the alveolar cleft. ILB is the most lateral border of the alveolar cleft. IMP was placed 8 mm in palatal direction from landmark IMB. ILP was placed 8 mm in palatal direction from landmark ILB. (C) Three-dimensional model illustrating the segmented cleft defect.

*Abbreviations*: SMB, superior medial-buccal; SLB, superior lateral-buccal; SMP, superior medial-palatal; SLP, superior lateral-palatal; IMB, inferior medial-buccal; ILB, inferior lateral-buccal; IMP, inferior medial-palatal; ILP, inferior lateral-palatal.

A total of eight landmarks were defined to determine the cranial, caudal, and palatal limits of the alveolar cleft. Figure 1A-C shows the landmarks in the cleft alveolus and palate defect in a schematic overview.

The most cranial part of the cleft was defined as the lowest part of the piriform aperture at the nonaffected side viewed from the coronal CBCT-slice, showing the mesial side of the first upper molar at the nonaffected side. Four landmarks were manually placed in the axial CBCT-slice of this point. The buccal outline of the cleft defect is a clearly defined anatomical landmark in the axial plane of the lowest part of the piriform aperture of the unaffected side. For this reason, landmark SMB was placed at the most medial-buccal point and landmark SLB was placed at the most lateral-buccal point of the alveolar cleft.

In earlier volumetric research on alveolar cleft grafting, the palatal part of the bone defect showed the largest interobserver variability.^7,9^ This might be due to the fact that the bony boundary of the cleft defect on the palatal side is not defined if the defect is ending in the hard palate. However, bony restoration of the palate in patients with cleft lip and palate has clinical relevance. Therefore, this study only used the relevant part of the palatal portion, that is, the palatal part of the defect belonging to the alveolar process. In the current literature, this part is not defined yet.

Since the palatal boundary of the alveolar cleft defect is hardest to assess, this method choses a more pragmatic approach to establish reliable and reproducible results. Previous research showed a minimal maxillary alveolar ridge width of 8 mm at the location of the canine.^10^ Therefore, the region of interest in this study used the first 8 mm of the alveolar process and leaves the palatal surface located dorsally of this 8 mm out of the region of interest. As such, landmarks SMP and SLP were manually placed 8 mm in palatal direction from landmark SMB and SLB, respectively, perpendicular to the axial plane of landmark SMB and SLB (Figure 1A).

The most caudal part of the cleft was defined as the cement-enamel-junction of the central incisor located medially of the cleft defect. In this axial CBCT slice, four landmarks were manually placed. Landmark IMB was placed at the most medial-buccal point of the defect and landmark ILB was placed at the most lateral-buccal point. Landmark IMP and ILP were manually placed 8 mm in palatal direction from landmark IMB and ILP, respectively (Figure 1B).

### Volume Calculation

**Figure 2.**
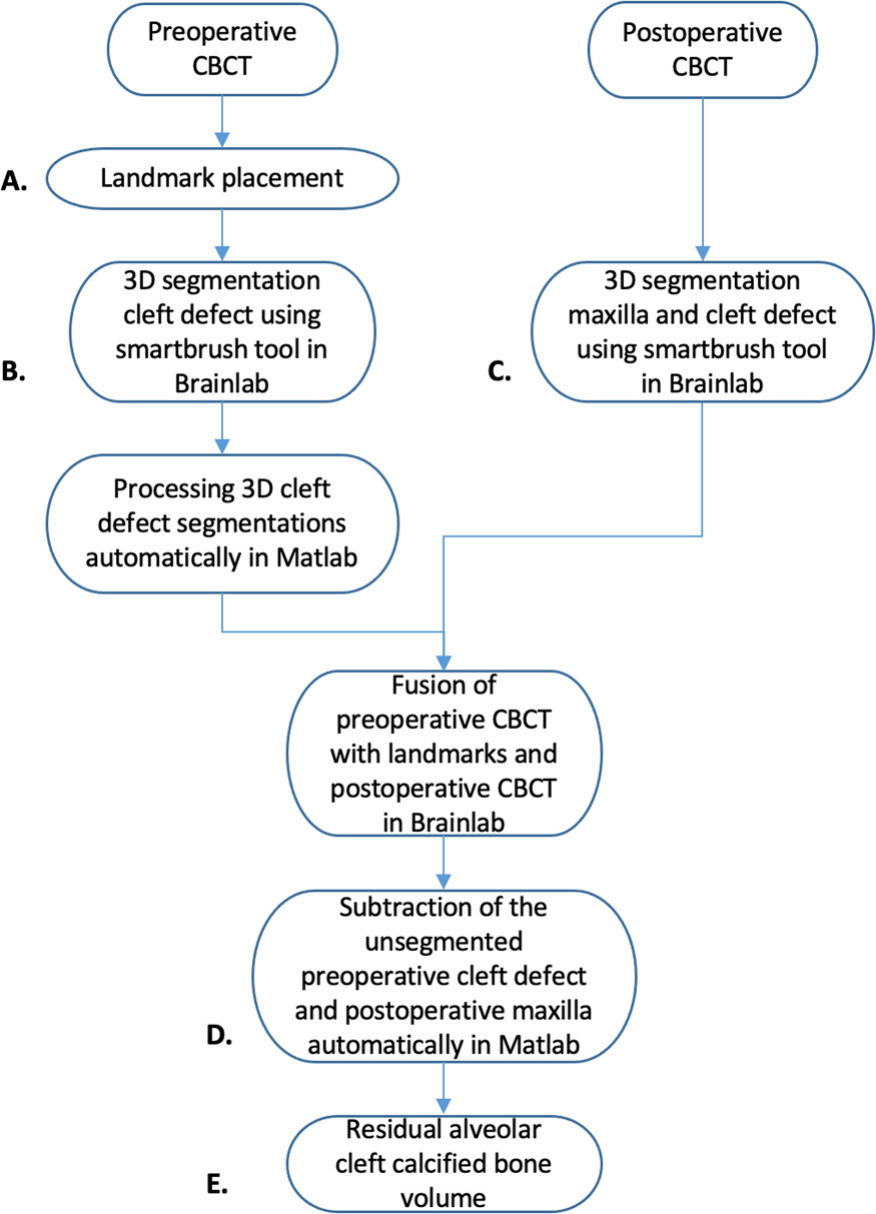
Schematic representation of the cleft volume calculation process. The process of volume calculation consists of landmark placement and segmentation, processing of the 3D segmentations, and calculation of the cleft volume. The letters A, B, C, D, and E are corresponding with Figure 3A-E.

The process of volume calculation consists of the following steps: landmark placement and segmentation, processing of the 3D segmentations, and the calculation of the cleft volume. For a schematic representation of the cleft volume calculation process, see Figure 2.

#### Landmark Placement and Segmentation

**Figure 3.**
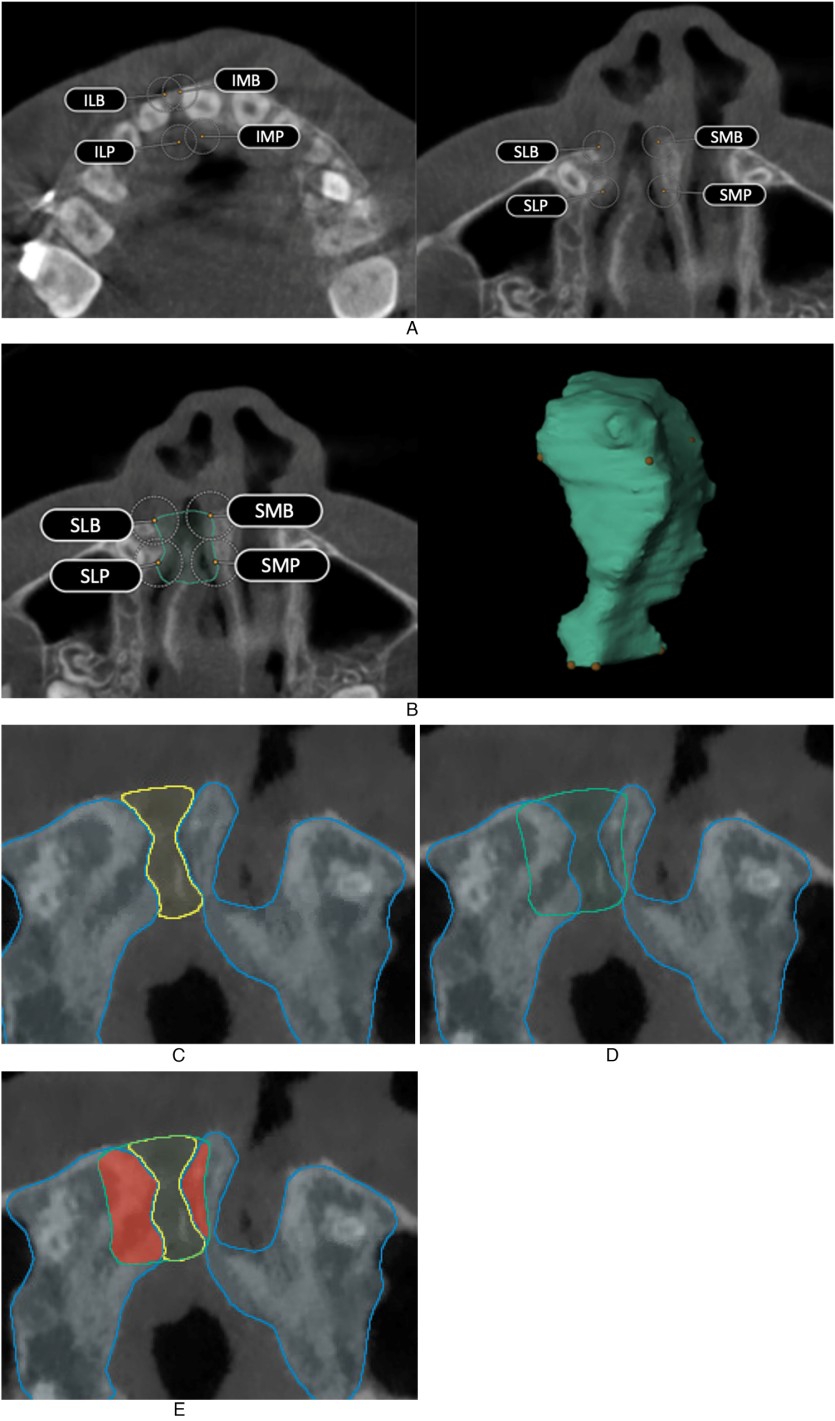
Cleft volume calculation process. (A) Landmark placement in the preoperative CBCT scan using BrainLab Elements. (B) Segmenting the preoperative cleft defect using the smartbrush tool. (C) Segmenting the postoperative maxilla and cleft defect. (D) Subtraction of the unsegmented preoperative cleft defect and postoperative maxilla automatically in Matlab. (E) Smoothened area between the lines shows the residual alveolar cleft calcified material.

The eight landmarks were manually placed in the preoperative CBCT scan using BrainLab Elements (Figure 3A). Additionally, the cleft defect in this preoperative CBCT scan was segmented using the smartbrush tool (Figure 3B). This tool was able to semi-automatically define the medial and lateral outline by following threshold values of alveolar bone. The segmentation of the palatal outline was initially performed by using the smartbrush tool beyond the palatal limits of the defect. Subsequently, subtraction of the overfilled area was executed after calculation of the palatal landmarks. The buccal outline was manually segmented. In order to determine the interobserver reliability, both the landmark placement and segmentation were performed by two independent observers (CS and NJ).

#### Processing of the 3D Segmentations

The landmarks and 3D segmentations of the alveolar cleft were exported for each observer. Further processing of the 3D was performed in Matlab (MATLAB 2020a, The MathWorks, Inc.), where the eight landmarks were used to define the cranial, caudal, and dorsal limits of the cleft defect.

Cranial limits were defined by a plane of the most cranial four landmarks of the defect (landmarks SMB, SLB, SMP, and SLP). Caudal limits were defined by a plane of the most caudal four landmarks of the defect (landmarks IMB, ILB, IMP, and ILP). The dorsal plane was defined by the mean plane of the most dorsal four landmarks of the defect (landmarks SMP, SLP, IMP, and ILP). These three planes were used to crop the 3D segmentations of the cleft defect. No cutting planes were defined on the buccal, medial, and lateral sides of the defect.

#### Calculation Cleft Volume

In the postoperative CBCT scans, the maxilla and the cleft defect was semi-automatically segmented using BrainLab smartbrush tool (Figure 3C). Thereafter, the preoperative and postoperative CBCT scans were fused in BrainLab Elements. The preoperative CBCT includes the unsegmented preoperative cleft defects of each observer. Subsequently, fused scans were used to automatically subtract the unsegmented preoperative cleft defect from the maxilla of the postoperative scan (Figure 3D). In this subtraction, the eight landmarks to crop the 3D segmentations of the cleft were used. The bony region within the preoperative alveolar defect was defined as residual alveolar cleft calcified material (Figure 3E).

### Statistical Analysis

All CBCT scans were segmented separately by two authors (CS and NJ) and evaluated by automated software to determine the interobserver reliability. Both observers were trained prior to performing this study. The interobserver reliability of the segmentation of the cleft was assessed by calculating the Dice-coefficient between the segmentations of the clefts based on the preoperative scan. The Dice-coefficient values the quality of image segmentation based on spatial overlap. This value is valid for reproducibility and accuracy in image segmentation, contrary to kappa statistic which is a chance-corrected measure of agreement between the segmentations.^11^

Similarity between the segmentations of the observers was calculated: a value of 0 represents no overlap of segmentations and a value of 1 represents complete overlap of segmentations. A good overlap occurs when the Dice-coefficient is >0.7 to represent a good interobserver reliability.^11,12^

Statistical analysis was performed using SPSS software for Windows (IBM SPSS Statistics, Version 26.0.0.1). The mean and standard deviation of the preoperative and postoperative defect sizes were calculated, as well as the residual alveolar cleft calcified material, given as the percentage of filled bone in the original cleft.

## Results

### Subjects

A total of 12 patients with unilateral cleft lip, alveolus, and palate were investigated. All patients were treated between June 2018 and July 2020. Mean age at the time of alveolar bone grafting was 10 years (range 9-11). Population consisted of 7 males and 5 females.

### Three-Dimensional Assessment Method

For reproducibility, standardized landmarks are required to calculate the volume of the alveolar cleft defect. Eight landmarks were defined: SMB, SLB, SMP, SLP, IMB, ILB, IMP, and ILP. The buccal landmarks could be appointed manually, since the buccal outline of the defect is clearly anatomically defined. The palatal landmarks were placed 8 mm in dorsal direction of the buccal landmarks. The most cranial plane was defined by SMB, SLB, SMP, and SLP. The most caudal plane was defined by IMB, ILB, IMP, and ILP. The palatal outline was defined by the mean plane of the four dorsal landmarks: SMP, SLP, IMP, ILP. The analysis was done twice by independent observers (CS and NJ).

### Interobserver Reliability

Mean Dice-coefficient for the segmented preoperative alveolar cleft defect was 0.81 (sd 0.03). The mean differences in volumetric measurements between observers was 0.07 cm^3^ (sd 0.05 cm^3^).

### Bone Volume

Mean preoperative alveolar defect size was 0.80 cm^3^ (sd 0.39 cm^3^). The mean postoperative defect size was 0.26 cm^3^ (sd 0.16 cm^3^). The mean percentage of the preoperative defect filled with grafted bone after surgery was 66.7% (sd 21%).

## Discussion

Several studies have evaluated SABG in patients with cleft lip, alveolus, and palate. Assessment of the volumetric cleft volume is difficult due to undetermined landmarks in the alveolar cleft defect.^6,13,14^ The most unreliable anatomical landmark of the cleft defect is the palatal plane.^9,15^ To avoid a standardized definition of the palatal plane, Du et al^16^ and Liu et al^17^ calculated the cleft volume defect by mirroring the alveolar bone of the nonaffected side using software. Limitation of these methods is that proper mirroring often is not possible due to the asymmetry of the maxillary arch caused by compression of the maxillary fragment lateral of the alveolar cleft.

Linderup et al described a method by standardizing the anatomical boundaries of the alveolar bone defect.^9^ In their method, the palatal margin was manually segmented on the basis of the contours of the contralateral alveolar bone. For intraobserver and interobserver reproducibility, Pearson correlation coefficients were *r* > 0.9849 and *r* > 0.8784, respectively, indicating excellent reproducibility. Despite the good reproducibility, limitation of this method is the inapplicability in cases of asymmetry of the contralateral maxillary arch. To overcome this problem, in our study the palatal margin was automatically defined as the mean plane of the most dorsal four landmarks of the defect. Our study showed a Dice-coefficient of 0.81 (sd 0.03 cm^3^), this value indicated that this three-dimensional method of assessing the unilateral cleft was reliable. A Dice-coefficient greater than 0.7 is considered as a good interobserver reliability.^11,12^

The placement of the palatal landmarks 8 mm in dorsal direction of the buccal landmarks was based on the minimal maxillary alveolar ridge width at the location of the canine. To reproduce the alveolar cleft defect, a standardized palatal outline is required. When applying this method in patients with a maxillary alveolar ridge width larger than 8 mm, the alveolar defect could be inaccurate and underestimated. This is a limitation of this assessment method. Further studies need to be carried out to evaluate the correlation between clinical success and the percentage of the postoperative filled bone grafting material in the standardized cleft defect.

Chen et al (2018) used a subtraction method in which customized boundaries of the alveolar bone defect were drawn automatically by using the “draw” function of 3D Mimics software.^18^ Reproducibility of these boundaries were not measured. In the present study, the medial and lateral limits of the cleft defect could be semi-automatically defined by using the smartbrush function in BrainLab. Thereby, eight landmarks were standardized to increase the reproducibility. Only the buccal border of the defect was manually defined. This may be a limitation. However, in segmenting the buccal margin of the maxillary arch no major differences were found between observers to reproduce this boundary, a Dice-coefficient of 0.81 (sd 0.03 cm^3^) was achieved.

Additionally, existing reliable methods seem time consuming and require a significant amount of technical knowledge. This finding was encountered using the previous semi-automatic segmentation protocol of our study group.^7^ Including the standardized landmarks into automated segmentation software could improve the practical use of this assessment method in the future. The current assessment method shows an easy-to-use, semi-automatic segmentation protocol, with accurate assessment of the cleft defect.

This study used CBCT scans for both accuracy and the low radiation dose. Current literature demonstrates that CBCT is applicable and accurate for volumetric cleft measurement methods. Despite the variation in field of view and voxel size, CBCT scans gave an overall high reliability.^19,20^

Calculating the peroperatively administered amount of bone grafting material may not be indicative for having a successful bone grafting treatment. Our study group does not consider it relevant to assess the amount of grafted volume directly after surgery. Alveolar cleft defects that are overfilled will generally show more resorption than defects that do not show overfilling and probably this is without clinical relevance. Additionally, there is an intrinsic osteoregenerative potential of the reconstructed defect that works in favor of alveolar cleft defects in which too little grafting material has been applied.^21^ The residual alveolar cleft calcified material percentage after SABG is 66.7% in this study. This is in accordance with previous findings.^22,23^ However, success of bone grafts was not within the scope of the present study.

This volumetric assessment method could be used to accurately and easily compare different cleft grafting materials or quantify operation techniques. However, clinical outcome parameters, such as eruption of the canine and lateral incisor, an uninterrupted arch and a closed oronasal communication, are as important to determine the success of the alveolar bone grafting treatment.

## Conclusion

In conclusion, this study demonstrates a reliable and practical semi-automatic three-dimensional volumetric assessment method for unilateral clefts using CBCT. Determination of cleft volume by using anatomical and standardized landmarks combined by three-dimensional medical image processing software provides a practical and reproducible assessment of the volumetric outcomes after SABG.

## References

[1] Stone C . Cleft lip and palate: etiology, epidemiology, preventive and intervention strategies. Anatomy Physiol. 2013;04.

[2] Boyne PJ Sands NR . Secondary bone grafting of residual alveolar and palatal clefts. J Oral Surg. 1972;30(2):87‐92. 4550446

[3] Bergland O Semb G Abyholm FE . Elimination of the residual alveolar cleft by secondary bone grafting and subsequent orthodontic treatment. Cleft Palate J. 1986;23(3):175‐205. 3524905

[4] Kindelan JD Nashed RR Bromige MR . Radiographic assessment of secondary autogenous alveolar bone grafting in cleft lip and palate patients. Cleft Palate Craniofac J. 1997;34(3):195‐198. 9167068 10.1597/1545-1569_1997_034_0195_raosaa_2.3.co_2

[5] Witherow H Cox S Jones E Carr R Waterhouse N . A new scale to assess radiographic success of secondary alveolar bone grafts. Cleft Palate Craniofac J. 2002;39(3):255‐260. 12019000 10.1597/1545-1569_2002_039_0255_anstar_2.0.co_2

[6] De Mulder D Cadenas de Llano-Pérula M Jacobs R Verdonck A Willems G . Three-dimensional radiological evaluation of secondary alveolar bone grafting in cleft lip and palate patients: a systematic review. Dentomaxillofac Radiol. 2018;48(1):20180047. 29947253 10.1259/dmfr.20180047 PMC6398910

[7] Janssen NG Schreurs R Bittermann GKP , et al. A novel semi-automatic segmentation protocol for volumetric assessment of alveolar cleft grafting procedures. J Craniomaxillofac Surg. 2017;45(5):685‐689. 28336322 10.1016/j.jcms.2017.02.018

[8] Amirlak B Tang CJ Becker D Palomo JM Gosain AK . Volumetric analysis of simulated alveolar cleft defects and bone grafts using cone beam computed tomography. Plast Reconstr Surg. 2013;131(4):854‐859. 23542257 10.1097/PRS.0b013e3182818e4f

[9] Linderup BW Küseler A Jensen J Cattaneo PM . A novel semiautomatic technique for volumetric assessment of the alveolar bone defect using cone beam computed tomography. Cleft Palate Craniofac J. 2015;52(3):e47‐e55. 25706336 10.1597/13-287

[10] Cho HJ Jeon JY Ahn SJ , et al. The preliminary study for three-dimensional alveolar bone morphologic characteristics for alveolar bone restoration. Maxillofac Plast Reconstr Surg. 2019;41(1):33. 31531306 10.1186/s40902-019-0216-2 PMC6726725

[11] Zou KH Warfield SK Bharatha A , et al. Statistical validation of image segmentation quality based on a spatial overlap index. Acad Radiol. 2004;11(2):178‐189. 14974593 10.1016/S1076-6332(03)00671-8 PMC1415224

[12] Zijdenbos AP Dawant BM Margolin RA Palmer AC . Morphometric analysis of white matter lesions in MR images: method and validation. IEEE Trans Med Imaging. 1994;13(4):716‐724. 18218550 10.1109/42.363096

[13] Stasiak M Wojtaszek-Słomińska A Racka-Pilszak B . Current methods for secondary alveolar bone grafting assessment in cleft lip and palate patients – A systematic review. J Craniomaxillofac Surg. 2019;47(4):578‐585. 30733132 10.1016/j.jcms.2019.01.013

[14] Choi HS Choi HG Kim SH , et al. Influence of the alveolar cleft type on preoperative estimation using 3D CT assessment for alveolar cleft. Arch Plast Surg. 2012;39(5):477‐482. 23094242 10.5999/aps.2012.39.5.477 PMC3474404

[15] Kochhar AS Sidhu MS Prabhakar M , et al. Intra- and interobserver reliability of bone volume estimation using OsiriX software in patients with cleft lip and palate using cone beam computed tomography. Dent J (Basel). 2021;9(2). 10.3390/dj9020014 PMC7911213 33499043

[16] Du F Li B Yin N Cao Y Wang Y . Volumetric analysis of alveolar bone defect using three-dimensional-printed models versus computer-aided engineering. J Craniofac Surg. 2017;28(2):383‐386. 28045819 10.1097/SCS.0000000000003301

[17] Liu B Yin NB Xiao R , et al. Comparison of two methods for presurgical volumetric evaluation of alveolar cleft bone defects using computer-aided engineering. J Craniofac Surg. 2021;32(2):477‐481. 33704964 10.1097/SCS.0000000000006930

[18] Chen GC Sun M Yin NB Li HD . A novel method to calculate the volume of alveolar cleft defect before surgery. J Craniofac Surg. 2018;29(2):342‐346. 29239924 10.1097/SCS.0000000000004181

[19] de Rezende Barbosa GL Wood JS Pimenta LA Maria de Almeida S Tyndall DA . Comparison of different methods to assess alveolar cleft defects in cone beam CT images. Dentomaxillofac Radiol. 2016;45(2):20150332. 26648387 10.1259/dmfr.20150332 PMC5083958

[20] Zhou WN Xu YB Jiang HB Wan L Du YF . Accurate evaluation of cone-beam computed tomography to volumetrically assess bone grafting in alveolar cleft patients. J Craniofac Surg. 2015;26(6):e535‐e539. 26355988 10.1097/SCS.0000000000002034

[21] Hellquist R Skoog T . The influence of primary periosteoplasty on maxillary growth and deciduous occlusion in cases of complete unilateral cleft lip and palate. A longitudinal study from infancy to the age of 5. Scand J Plast Reconstr Surg. 1976;10(3):197‐208. 1053449 10.3109/02844317609012969

[22] Xiao WL Zhang DZ Chen XJ Yuan C Xue LF . Osteogenesis effect of guided bone regeneration combined with alveolar cleft grafting: assessment by cone beam computed tomography. Int J Oral Maxillofac Surg. 2016;45(6):683‐687. 26876144 10.1016/j.ijom.2016.01.013

[23] Janssen NG Schreurs R de Ruiter AP , et al. Microstructured beta-tricalcium phosphate for alveolar cleft repair: a two-centre study. Int J Oral Maxillofac Surg. 2019;48(6):708‐711. 30594478 10.1016/j.ijom.2018.11.009

